# Recent Insights into NIR-Light-Responsive Materials for Photothermal Cell Treatments

**DOI:** 10.3390/nano12193318

**Published:** 2022-09-23

**Authors:** Md Imran Hossain, Sitansu Sekhar Nanda, Subramanian Tamil Selvan, Dong Kee Yi

**Affiliations:** 1 Department of Chemistry, Myongji University, Yongin 17058, Korea; 2 Alpha Biomedical Pte Ltd., 21 Biopolis Road, Nucleos North Tower #02-03, Singapore 138567, Singapore

**Keywords:** cell behavioral control, inflammation control, photothermal therapy, bioactive materials, nanoparticles, metal nanoparticles

## Abstract

Controlling cells using photo-responsive materials is highly indispensable in the current biomedical sector. Considering the potential side effects of nanoparticles, it has become a challenge to control cells with photo-responsive materials. Recent studies have described several methods for controlling cell behavior using nanoparticles subjected to the near-infrared (NIR) laser light operating at the wavelength of 808 nm to 980 nm and at the power densities of 0.33 to 0.72 W·cm^−2^. The challenge here is the preparation of biocompatible nanoparticles for both in vivo and in vitro studies and understanding cell behavior with an external light source recommended for biological application. Earlier studies have well documented many approaches and associated mechanisms for controlling cell behavior and the interaction between nanoparticles, cells, and appropriate external light sources. In this review, various nanomaterials such as metal nanomaterials and carbon-based nanomaterials are compared systematically regarding the effects of controlling cell behavior and inflammation by studying their mechanisms, route of administration, dose, and adverse effects such as toxicity and the interaction of nanoparticles with a specific wavelength of the light. Future directions should focus on stable and efficient light-responsive materials with minimal cytotoxicity.

## 1. Introduction

Nanoparticles (NPs) are ultra-fine small particles with sizes ranging from 1 to 100 nanometers. Metallic NPs such as gold, silver, platinum, and palladium respond when the light is applied in vivo [1,2,3]. NPs can be synthesized in a variety of shapes and dimensions. This opens new possibilities for selectively targeting cancer cells, as well as other emerging photothermal therapies (PTT) [4]. Designing light-responsive NPs is indispensable, owing to their NIR light absorption, leading to the generation of heat, and paving the way for the thermal ablation of cancer cells. However, nanomaterials should exhibit strong light-absorbing properties for the transfer of optical energy into heat [5,6,7]. Changes in the particle size and shape of nanoparticles may affect the surface plasmonic characteristics, which allows us to tune the properties of the nanomaterials for different biomedical applications [8]. Conventional treatments to control cell behavior such as chemotherapy and radiotherapy have serious side effects, and they can damage healthy cells during the treatment period [9,10]. To overcome this issue and make the treatment selective to specific cells, the application of nanomaterials under the NIR light irradiation has been introduced. Nanomaterials can pass through abnormal cell membranes such as cancer cells where conventional materials have a larger size than the nanomaterials and are thus not able to pass through the cancer cells’ membranes. NIR light is more likely to penetrate in these scenarios with only mild damage compared to ultraviolet and visible light [11].

NPs with anti-inflammatory properties have been used for controlling autoimmune and inflammatory disorders such as rheumatoid arthritis [12]. For instance, NPs such as Au, Ag, and iron oxide (Fe_3_O_4_) nanohybrids have been well documented in the literature as carrier drugs for treating inflammation and arthritis [13,14,15,16,17]. Brown et al. demonstrated that AuNPs (gold nanoparticles) can be used to treat rheumatic diseases associated with inflammation [18]. Conversely, AuNPs were used as potential antiangiogenic agents with less toxicity [19,20]. Carbon-based nanomaterials such as single-walled carbon nanotube (SWCNT) or multiple-walled carbon nanotube (MWCNT) are potential nanomaterials for cell behavior control due to their optimum tunable properties. This review attempts to provide a comprehensive overview of the application and limitations of light-responsive nanomaterials for inflammation control and their associated cell behavior for the last 5 years.

## 2. Application of Nanomaterials to Control Cell Behavior under Light Exposure

**Figure 1:**
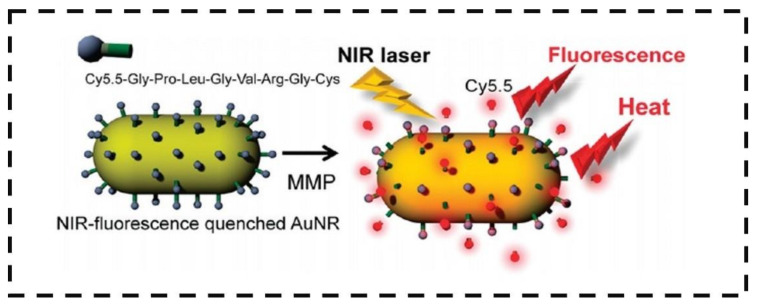
Schematic diagram of MMP-functionalized AuNRs for simultaneous imaging and photothermal therapy. Adapted with permission from Ref. [22]. 2010, American Chemical Society.

Selective cancer therapy comes from the background of treating cancer cells with nanomaterials and applying light to the NPs. The research on “photo-nano-therapy” has been dramatically increasing since 2010 [21]. PTT has two diverse levels: (1) active or passive tumor homing through engineered phototherapeutic agents such as NPs; (2) irradiating the diseased lesion without harming the normal cells by the controlled light application. Earlier, our group established the application of matrix metalloproteinase (MMP) sensitive gold nanorods (AuNRs) as an efficient photothermal agent for simultaneous cancer diagnosis (high NIR fluorescence after 60 min of injection) and therapy (enzymatic activity and damage to cancer tissue) (Figure 1) [22].

PTT allows for NPs to absorb light and generate heat, resulting in the thermal ablation of carcinoma cells, leading to cell death. Specifically, upon light exposure, an ideal photothermal agent should be capable of absorbing light and generating the reactive oxygen species (ROS) to induce cancer cell lysis [23]. In this review, we cover the photothermal application of Au, Ag, Pd, Pt, and carbon (C) NPs in dissimilar sizes and shapes.

### 2.1. Metal Nanomaterials

Surface plasmon resonance (SPR) is an interesting phenomenon occurring in metal NPs, due to the excitation of electrons in the metal surface layer by photons of the incident light [24]. When interacting with light, metal nanomaterials convert light into heat if their oscillation is resonant at the practical frequency of the light [25]. Our group studied photothermal therapy using AuNPs and has been receiving a great deal of interest among researchers because of its excellent optical properties and heat-absorbing capacity [26]. Several gold nanostructures such as AuNPs, AuNRs, gold nanocrystals, gold nanostars, and gold nanoflowers have existed in prior arts. AuNPs have been shown to destruct both cancer and bacterial cells. Our group has demonstrated the potential application of AuNRs for photothermal therapy [7,22,27,28,29,30,31,32]. We have summarized the NIR-light-responsive materials for photothermal cell treatments in Table 1.

Importantly, AuNRs have two characteristic optical absorptions, i.e., the transverse and the longitudinal, corresponding to the aspect ratio (i.e., length/diameter) of the rods. Thus, by tuning the aspect ratio of AuNRs, the SPR region could be shifted to the NIR region for PTT. Upon NIR light irradiation, AuNRs absorb light which is effectively converted into heat because the excited conduction band electrons decay to the ground state by releasing their energy. The NIR light provides the maximal penetration of light, up to 10 cm (breast tissue), depending on the tissue types due to relatively lower scattering and absorption from the intrinsic tissue chromophores [40].

Reducing the cytotoxicity by coating silica NPs on the AuNRs is one of the useful studies for optothermal cancer cell lysis. Earlier, we demonstrated that silica-coated negatively charged (−24 mV) AuNRs added to the HeLa (derived from the name Henrietta Lacks) cells showed minimal toxicity [41]. Another study from our group reported that Si-AuNRs have a 36.13% greater cell growth rate for MDA-MB-231 cells (human breast cancer cells) under the NIR laser irradiation than the normal incubator condition by enhancing the activity of heat shock protein (HSP) [29]. Our recent study demonstrated the photothermal application of copper–gold (Cu-Au) tripods for CT-26 cells (colon carcinoma cells) death under the laser irradiation of 633 nm wavelength, 150 mW/cm^2^ for 10 min [42]. Additionally, magnetic nanoparticles (MNPs)-conjugated AuNRs could generate a rapid photothermal effect and produce a bactericidal effect by enhanced magnetic separation [7].

Silver nanoparticles (AgNPs) have the potential thermal activity to control cell behavior effectively. Boca and co-workers reported that modified synthesis of chitosan-coated silver nano-triangles (Chit-AgNTs) showed effective photothermal activity against human non-small lung cancer cells (NCI-H460). In more detail, enough positively charged Chit-AgNT with the zeta-potential of +39 mV can provide enough surface charge to stablize the particles. Chitosan can be the alternative to biopolymers to make the NPs more stable and more biocompatible [33]. The efficiency of the conversion of light into heat is similar for both Au and AgNPs [43,44]. Liang and co-workers observed that spiky star-shaped Au/Ag NPs also have the potential to deal with cancer cells by using photothermal effects. They made fluorescein isothiocyanate (FITC)-labeled modified chitosan-coated Au/Ag NPs and used oral cancer cell (SAS) with 150 mW of NIR laser for a treatment period of 12 h to induce the ablation of the cancer cell compounded by the laser wavelength of 800 nm [45]. However, the antimicrobial role of AgNPs products is now a matter of interest. Properties such as biocidal, virucidal, localized surface plasmon resonance (LSPR), and anticancer activity make AgNPs more selective for modern biomedical applications [46]. Smaller particles that give higher cytotoxic effects impair large surface area. Because of the well-known shapes of AgNPs that can utilize the nanostructure in the biological field such as nanowire, nanorod, and nanoplate [47]. AgNPs, used as anti-angiogenesis in albino mice and LD50 (lethal dose 50): 3.5 µL/mL AgNP function as an anti-cancer material for MCF-7 breast cancer cells [48]. Sahu and co-workers reported that AgNPs have an anticancer effect on hepatic cells. They applied AgNPs on HepG2 (hepatocellular carcinoma) cells with a size of 20 nm and the dose was 1–20 µg/mL with an incubation time of 24 h under the NIR irradiation, finding that AgNPs affected the hepatic cells, though they had a cytotoxic effect [49].

Palladium nanoparticles (PdNPs) are widely known for their use in the green synthesis method. Ruiz and co-workers found that PdNPs synthesized by the green method with sizes of around 6 nm can accommodate the cancer cell cytoplasm. After applying the NIR laser, they showed the cell ablation effect on the cancer cells [50]. Ultrathin hexagon-shaped PdNPs with sizes ranging from 28 to 60 nm have excellent catalytic and plasmonic properties upon NIR laser [51]. In another study, Tang and co-workers suggested that high photothermal conversion of PdNPs has been found in the NIR at 808 nm wavelength. They also reported that the surface modification of the palladium nanosheets reduced the glutathione with sustained blood circulation and with a high accumulation rate in the tumor site [52]. They reported a higher conversion efficiency of PdNPs compared to typical gold nanorods and the efficacy of the photothermal conversion is 93.4% at a laser power of 808 nm, killing over 70% of cancer cells within 4 min. After modifying the surface of PdNPs with chitosan oligosaccharide, it shows improved biocompatibility [53]. PdNPs–Chitosan compounds can functionalize the RGD (arginylglycylaspartic acid) peptide, improving its accumulation in breast cancer cells and showing a therapeutic effect under an 808 nm laser [54]. Flower-shaped PdNPs embedded in chitosan/polyvinyl alcohol membrane with sizes ranging from 30 to 50 nm also have photothermal and wound-healing activity as reported in an article. Briefly, different concentrations of PdNPs were used, and 60 µg/mL of PdNPs rapidly went up to 56.5 °C and 6.25 µg/mL of PdNPs went to 33.7 °C [55]. Chaga Mushroom-derived PdNPs are useful for tri-modal anticancer therapy, controlled delivery of doxorubicin, and photothermal activity upon laser irradiation, while 40 µg/mL Chaga-PdNPs under 808 nm laser irradiation for 4 min can cause the cell ablation in HeLa cells [56].

Platinum nanoparticles (PtNPs) have become a scientific tool that is explored in various nanomedicine and biotechnological fields. Evidence has proved that photothermal therapies of PtNPs are highly selective for tumor ablation in both ways, such as singlet oxygen generation and the photothermal effect. Iron-conjugated PtNPs showed improved bioavailability on photothermal therapy by high NIR laser [57]. Folate functionalized 3-mercaptopropionic acid (FePtNPs) size of around 12 nm impacted the intercellular damage, which corresponded to the number of NPs causing necrosis in tumor cells in a proportional manner [58]. Trifolium-like platinum nanoparticles (TPNs) have been studied for their photothermal effects and it has been found that TPNs are effective for killing cancer cells followed by four hours of incubation and 808 nm NIR laser irradiation for 5 min. In vivo analysis of TPNs showed a clear reduction in tumor growth [59]. Peptide modification of PtNPs showed improved bioavailability and accumulation in mitochondria and PtNPs generate hypothermia in thermosensitive mitochondria, resulting in limiting tumor growth and severe damage to cancer cells. Protein-conjugated ultra-small PtNPs can specifically target mitochondria under the irradiation of 1064 nm laser by 5 min with a concentration of 32 µg/mL, 1.5 W/cm^2^ [60]. In one study, it is shown that injectable and degradable hydrogel-based PtNPs can be used for repeatable photothermal cancer therapy. Dex-Ald and dendrimer-encapsulated platinum nanoparticles (Dex-DEPts) can raise the temperature to ~65 °C upon 808 nm NIR irradiation within 3 min. Dex-DEPts hydrogel was maintained in the tumor for one week repeatedly and it was found to cause further tumor regression [61].

Moreover, tumor-specific nanoparticles-based photothermal therapy has prominent contributions in the field of cell behavior control. Xiong and co-workers developed a hybrid biomimetic membrane (IRM), indocyanine green (ICG)-loaded magnetic nanoparticles (Fe_3_O_4_-ICG@IRM) for photothermal immunotherapy. This prolongs the circulation half-life, biodistribution, and response in tumor-specific immunotherapy [34]. However, nano–bio interactions and bioprocessing are aspects to be considered for the internalization of nanoparticles into the cells. Hollow copper sulfide (CuS) and rattle-like iron oxide nanoflowers@CuS core-shell hybrids (IONF@CuS NPs) are effective in the cellular metabolism of the nano-sized metals without affecting the cell viability and oxidative stress [35]. Additionally, one-dimensional polycation-coated nanohybrids Fe_3_O_4_@Dex-PGEA composed of polysaccharide dextran showed excellent photothermal properties, cellular uptake, and rapid clearance [36].

### 2.2. Carbon-Based Nanomaterials

Carbon nanotubes (CNTs) are well-structured, hollow, graphite nanomaterials and many researchers are attracted to them for having different layers, such as SWCNT or MWCNT, and a tunable length. Because of their high mechanical strength and extended surface area and low-weight molecules, researchers are exploring their potential for biological and biomedical applications though it has cytotoxic effects [62,63]. Growing the cells for tissue regeneration and varieties of the targeted drug delivery, diagnostic and gene transfection are being studied in this field. Although CNT has specific biomedical and biological applications, it has severe toxicity toward human health and the surroundings. Dumortier et al. noted that functional carbon nanotubes have deep adverse effects on immune cells and reported few deaths [64]. For controlling the enzymatic activity, MWCNTs/SP is competent as stated by Song et al. [65]. The enhanced permeability and retention effect (EPR) and the high levels of intrinsic absorption properties make the carbon NPs captivating agents for photothermal therapy [66]. Owing to their EPR and magnificent underlying properties, the discriminatory heating of carcinogenic tissue with or without anticancer drugs is desirable to administer selectively occurring in photocoagulation accompanied by cell death, scaling down the dimensions of the carcinogenic tissue, or complete elimination of the selective tissue [67]. There are several reasons for choosing the biological treatment of carbon nanotube: First, the proper surface modification of the carbon nanomaterials makes the molecules protected from the attack of the immune system [68]. We correlated specific treatment methods by using different nanomaterials under different NIR laser conditions in Table 1. Second, carbon NPs have prominent light absorbance in the NIR, having superior tissue penetration ability [69]. SWCNTs were the first carbon NPs utilized as a photothermal agent and the administration of SWCNTs was based on intratumoral injection, and intravenous injection [37,70].

**Figure 2:**
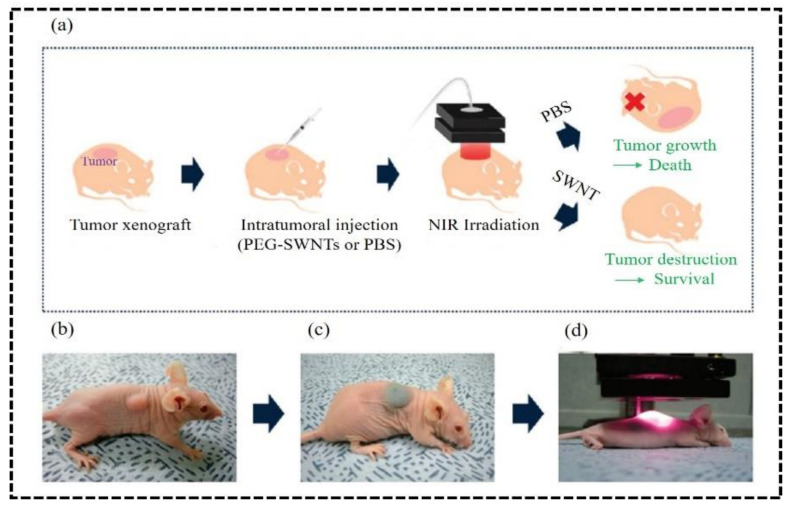
Photothermal treatment of PEG-SWCNTs has been shown here in mice. (**a**) schematic representation of the whole treatment procedure in mice; (**b**) mice bearing KB tumor cells; (**c**) photograph of the mice after administration of PEG-SWCNTs; (**d**) Irradiation of NIR on the tumor site (808 nm, 76 W/cm^3^) for 3 min. Adapted with permission from Ref. [71]. 2009, American Chemical Society.

Chao et al. indicated advanced administration of carbon nanotube to the tumor metastases in sentinel lymph nodes (Figure 2) [71]. Most cancer deaths are associated with metastasis spread. So, it is decisive to destruct the cancer cell from the dominant level. In a study, researchers found that the irradiation of both primary and secondary tumors by photothermal heating prolonged the mouse survival, in contrast to the only primary tumor [72]. However, optimum renal clearance of the nanoparticles is a challenge in the way of achieving low systemic toxicity. Zeng and co-workers developed ultrasmall polypyrrole nanoparticles (Ppy NPs) of the size ~2 nm which have excellent photothermal conversion efficiency from 33.35% to 41.97% with efficient renal clearance [73]. The introduction of graphene-based nanocomposites as 4D-printed materials for on-time and position shape transformation under the NIR irradiation contributes potentially to the biomedical field [74]. Additionally, we have summarized the size-dependent biocompatibility or toxicity in Table 2 [38,39,75,76,77,78,79].

## 3. Functions of Nanomaterials to Control Inflammation

**Figure 3:**
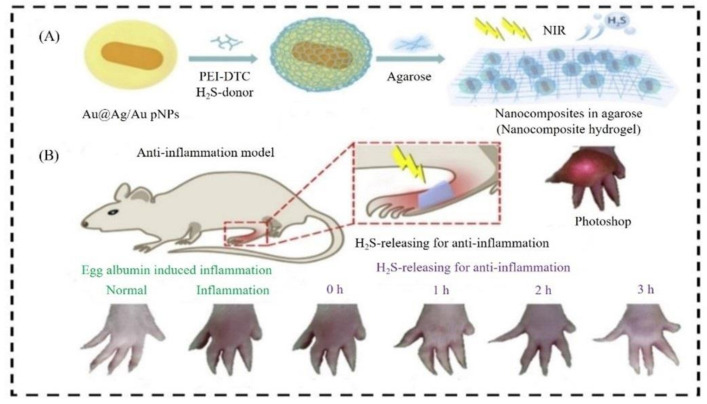
Assessment of anti-inflammation of nano-hydrogel in the systemic circulation. (**A**) Illustration of designing nanocomposite hydrogel (**B**) Rat toes in different conditions in a different time interval. Adapted with permission from Ref. [80]. 2020, Elsevier.

The stage of the inflammation or inflammatory reactions revolves around the adsorption of the proteins, for example, albumin, fibrinogen, fibronectin, and vitronectin. A study revealed that polyethyleneimine dithiocarbamate-based H_2_S donor, the photothermal nanomaterial can control the H_2_S release to cure inflammation (Figure 3) [80]. The damage originated from biomaterials implantation, and surface-anchored proteins engage neutrophils, and cause the active part of the resident mastocyte, culminating in the acute inflammatory response [81]. Inflammatory responses to nanomaterials depend on the size of the NPs. Fibrinogen-mediated activation of Mac-1 receptor is increased when the particle length is less than 20 nm [82]. The effect of the size of NPs on the inflammation site is also being recognized by comparing 75 nm and 200 nm polymorphonucleocytes (PMN) NPs, 75 nm PMN evoked limited engagement in the bronchial alveolar lavage fluid instead of 200 nm. Additionally, rod-shaped particles with the size of 100 nm have greater cellular uptake than other shapes of the NPs such as cube-shaped, cylindrical particles [83]. NPs may induce inflammatory responses independently of cell interaction. Surface radical electron-induced ROS-generating capacity in the presence of suitable substrates such as platinum nanoflower. In a study, it is mentioned that platinum nanoflower produces more ROS levels than platinum multi-pod [84]. NPs were reported as a vehicle to deliver anti-inflammatory drugs and produced biological responses. Poly (lactic-co-glycolic acid) (PLGA)/Polyethylene glycol (PEG) and Col IV peptides can productively transport drugs mimicking anti-inflammatory mediators [85,86]. It involved several forms of NPs in the application to the inflammation site, for example, nano-porous scaffolds, nanopatterned surfaces, nanofibers, and CNT [87,88,89,90].

Iron oxide nanoparticles (IONs) can be synthesized by using chemical compositions such as Fe_3_O_4_ or ƴ-Fe_2_O_3_, but the most familiar type of IONs is the non-stoichiometric combination of the two. The magnetism behavior of the iron oxide can be determined by the size of the particles [91]. When iron oxide nanohybrids are used as contrast agents such as in MRI (magnetic resonance imaging) agents, they are coated with biocompatible materials to reduce cytotoxicity and heighten the contrast property [92]. After applying an external alternative magnetic field, iron oxide leads to the production of heat, and it can be termed magnetic hypothermia. It is noted that after applying the high-power light source, the thermal output caused by the iron oxide nanocrystal is enough to induce temperatures above 42 °C; as we mentioned earlier in this review, 42 °C can cause necrosis [93]. The reason for making iron oxide nanohybrids is because they low absorption in the NIR and therefore, apparently, a poor photothermal effect. The external magnetic environment inside the body can limit NPs movement, known as magnetic targeting. Magnetic targeting is the method where the other functions of magnetization of the NIR light can control the nanohybrid to be guided to the tumor or as a contrast agent, such as an MRI agent or real-time monitoring of the tumor or treatment. Instead of iron oxide, AuNPs have been adopted for a long time, and are compatible with photothermal applications.

**Figure 4:**
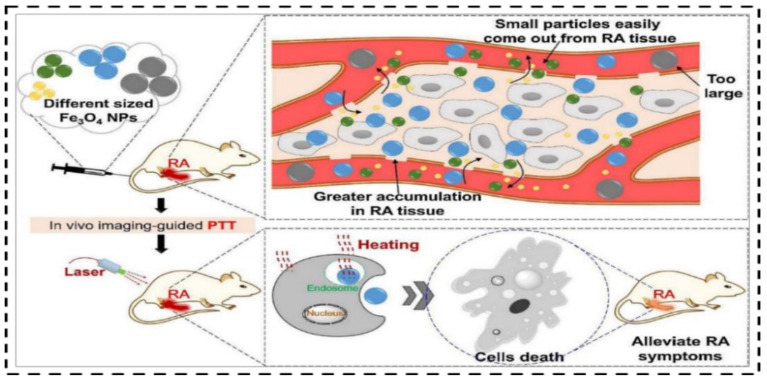
Schematic illustration of the in vivo imaging-guided photothermal effect of varied sizes of IONs for rheumatic arthritis with mentioning IONs accumulation in the affected tissue. Adapted with permission from Ref. [96]. 2018, Elsevier.

LSPR understanding of the gold NPs is dependent on the diameter of the particle: for example, the LSPR peak for the 10 nm particle is 520 nm and 100 nm particle is 580 nm. However, it is feasible to tune the LSPR wavelength of the NPs close to the NIR. This is a way of controlling different AuNPs via, for example, gold nanorods, nanocages, and nano-shells, which are being applied as photoacoustic imaging agents in tumor models in vivo because of their tunable LSPR around the NIR [94]. The traditional use of gold-IONs NPs is done in the same way that iron oxide cores, surrounded by gold nanoshells, are used for MRI and PTT. This followed two other strategies to prepare gold nanoshell-coated IONs. The simple method for coating the gold and iron oxide NPs is to use a silica layer or polymeric layer before biological application [95]. Zhang and co-workers found that IONs with sizes ranging from 70 to 350 nm have an excellent effect on photothermal conversion. Large particles showed superior photothermal conversion and the small particles were more quickly engulfed by inflammatory cells than their large counterparts (Figure 4) [96].

**Figure 5:**
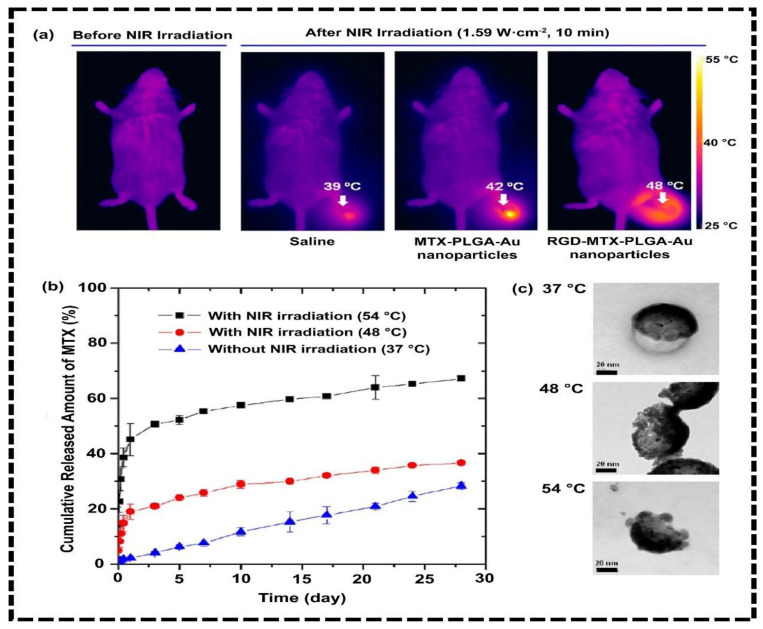
(**a**) Thermal images of different mice treated in different conditions; (**b**) MTX release profile with NIR, without NIR. (**c**) Transmission electron microscopy (TEM) images of MTX release experiments in different temperatures without and with NIR irradiation. Adapted with permission from Ref. [100]. 2013, American Chemical Society.

In an experiment, the rat (Wistar rat) was injured by the laser (3 W/cm^2^, 5 min), then magnetically targeted mesenchymal stem cells (MSCs) were incorporated for the wound therapy. The improvement of the anti-inflammatory ability has been found during the treatment of NP-labeled MSCs in rats. This is potential evidence for the future to boost the work regarding the use of MSCs to heal wound sites [97]. Tang and co-workers demonstrated the ability of SDIO (iron oxide coated with dextran sulfate) targeting scavenger receptor class A to visualize microglia actively, which implied brain inflammation. Functional IONs were found to be safe and internally effective by activating microglia for in vivo and in vitro studies. They found that IONs coated with dextran sulfate are a favorable contrast agent for MRI. IONs have the potential for multiple roles to visualize activated microglia in inflammation [98]. Methotrexate (MTX) is also used for treating rheumatoid arthritis (RA) but there are some complications regarding MTX. Long-time use of MTX can cause hepatitis and bone marrow suppression [99]. This is the reason researchers tend to focus on nanomaterials to reduce the toxicity for treating RA and inflammation. Lee and coworkers developed MTX-PLGA-Au NPs conjugated with amino acids on the surface of the gold-half shell, in which the targeting moiety was arginine-glycine-aspartic acid that provided anti-inflammation activity with lower toxicity (Figure 5) [100].

In another investigation, Vadim et al. explained the biochemical mechanism of AuNPs-dependent downregulation of IL-1β-promoted inflammatory responses. They found the potential therapeutic response of AuNPs during treating IL-1β-dependent autoimmune disorders [101]. Another study reported that AuNP@PEG@BSA.Ru was enough to produce a greater cell effect at the tissue repair and inflammatory level because of having rapid cell uptake that increases carbon monoxide inside the target cell to produce a potential response [102].

**Figure 6:**
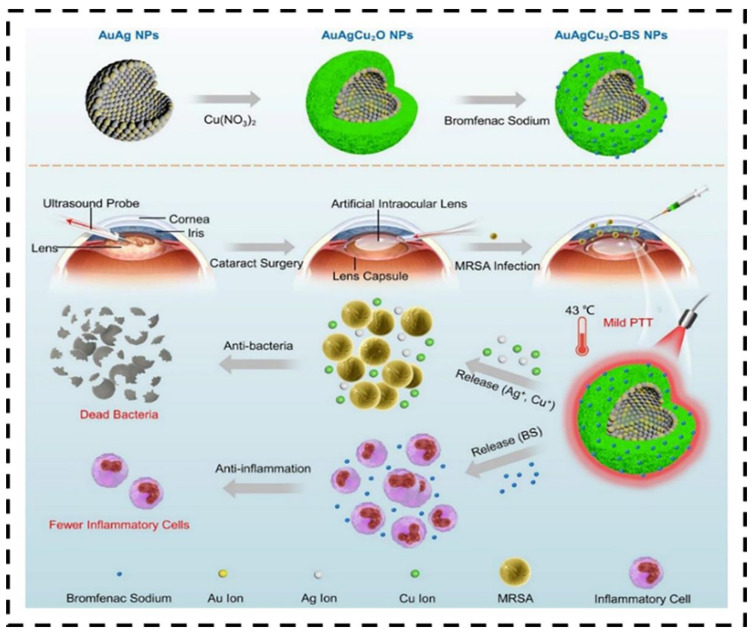
The illustration shows the treatment of AuAgCu_2_O-BS NPs to the endophthalmitis after cataract surgery. Adapted with permission from Ref. [106]. 2020, Ivyspring International Publisher.

Precisely, the immune system works to eliminate illicit factors and helps to organize the tissue and restore it [103]. During the inflammatory response, the damaged site feels discomfort. Failure in the quick response of the immune system can guide the progression to temporary or permanent inflammatory disorders [104]. Evidence has shown that AgNPs synthesized from plants have anti-inflammatory activities by stimulating the yield of cytokines and acting as capping agents. El-Rafie et al. found that *Terminalia* species-mediated incorporated AgNPs that contain flavonoids, phenolics, proteins, and polysaccharides that provide deleterious effects on free radicals and powerful anti-inflammatory effects [105]. Modified drugs such AuAgCu_2_O-bromfenac sodium (BS) administrated to the anterior chamber as IV injection under the NIR laser irradiation, displaying anti-inflammatory effects by the controlled release of nanomaterials (Figure 6) [106].

## 4. Mechanisms of Cell Behavioral Control Using Nanomaterials under Light Irradiation

**Figure 7:**
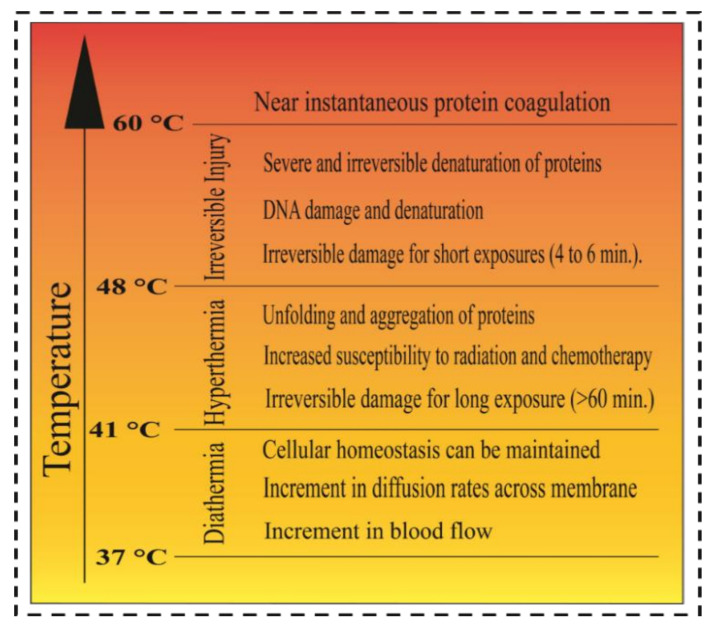
Different temperature states where the cells react differently leading to denaturation of the protein. Adapted with permission from Ref. [113] (Redrawn). 2014, Royal Society of Chemistry.

The study of nanomaterials in the existence of light may have a strange effect in both internal and foreign surroundings on the tissue. Cognizing the cooperation between light and nanomaterials in both surroundings is very essential to understanding the mechanisms such as thermal effect, catalysis effect, or/and other changing the enzyme activity [107,108]. Our recent study showed several ways of controlling cell behavior using mechanochemical cues [109]. NIR is counted as a beneficial process for diagnosis with selective therapy for deep penetration in the tissue. Recent papers described that NIR light can penetrate the deep lesions of carcinoma tissue more than in normal tissues: for example, the wavelength of 630 nm penetrates the tissue of a normal brain up to around 0.9 mm, and in contrast, the penetration to lung cancer tissue is about 1.6 mm [110]. Besides the wavelength, the intensity of NIR can play a vital role in the interaction between tissue and light. Concerning NIR light wavelengths ranging from 808 nm and 980 nm, the safe laser intensity for normal cells determined by the American National Standard for safety is around 0.33 W·cm^−2^ for the 808 nm NIR laser power and around 0.72 W·cm^−2^ for 980 nm of wavelength NIR laser [111,112]. Due to the temperature increment, the thermal treatment of the carcinogenic cell can be classified into three special categories: irreversible injury approaches, hyperthermia strategies, and diathermia practices. Irreversible injury approaches are high-temperature treatments for the cell (Figure 7) [113].

Above 48 °C, treatment for a few minutes can activate the cell death, resulting in necrosis. When the temperature is more than 48 °C and applied to the cell, the effect is serious on the cell and non-reversible. This treatment is the most effective for cancer cells, but it also affects the healthy cell, lacking selectivity, so this treatment needs to be more selective [114,115].

In hyperthermia treatments, the temperature range in these treatments are 41 °C to 48 °C, it is also called the clinically appropriate temperature. The fundamental mechanisms of hyperthermia involved temporary cell inactivation and aggregation of the proteins. Usually, it is used with other materials as a combination therapy with other cancer treatments [116]. The increase in ROS level inside the cell results in oxidative stress in the cell. Hyperthermia treatment could cause protein denaturation by lowering the production of HSP. Diathermia treatments are moderate heating treatments with temperature increments up to 41 °C. It is beneficial for several health effects such as increment in blood flow, and diffusion rate increment throughout the ion channels. Increasing the temperature inside the tumor can increase the blood flow between the tumors; thus, is improves the chemotherapy. An increment in blood flow can also enhance the migration of immune cells to the target [117].

The physical contour of the extracellular matrix microenvironment may also control cell behavior, including micro- and nanoscale contour features. For instance, properties of the cell membrane affect the particles to enter the cells such as collagen fiber diameters ranging from 0.5 to 3 μm wide and the pore size diameter is 1–5 μm where the size of the cell is 1–100 μm [118]. Because of the pore size of the cell membrane, NPs became more widely applicable material to treat cells. The modulation of extracellular matrix (ECM) controlled various cell behavior, including cell proliferation, differentiation, apoptosis, protein assembly, and disassembly function [119]. The stacking and clustering of integrin receptors to the extracellular matrix are involved in cell addition. The integrin signal determined the cell shape and structure. La Pointe et al. said that by regulating the integrin signal, endothelial cell proliferation, differentiation, and apoptosis are controlled [120]. When mechanical and biochemical changes occur, cells are used to respond to it by ECM through the signaling communication between integrin and the actin cytoskeleton. Our recent study showed the application of AuNRs coated with peptides to inhibit the activation of lysine-specific demethylase [121]. Cell fate, such as cell division, differentiation, contractility, and motility, relies on mechanical force. Mechanotransduction, the term based on the conversion of the mechanical properties to the cellular response, is an expanding field of science [122]. Tseng et al. demonstrated that it gave many individual magnetic nanoparticle doses to single cells that are arranged by a uniform pattern with arrays of magnetizable ferromagnetic materials. They found that localized NPs induce mechanical tension in a cell producing a cellular response in both biological response and biochemical processes. When the micromagnet is magnetized by any permanent magnet, it generates the potential minima that are rapidly localized to the NPs inside the cell [123]. In the state of hypoxia in the tumor, it has a high rate of stimulating neovascularization and metabolism, which improves photodynamic therapy. Recently, Jun and co-workers developed a bismuth selenide nanoparticles (Ab-PEG-Bi_2_Se_3_) material that gives higher absorption in the near infrared-II window with deep tissue penetration. This material has the potential to cause imaging-guided hyperthermia in the tumor microenvironment [124]. In the early apoptosis phase, the cell surface presents phosphatidylserine, which leads cells to apoptosis. Compared to the PTT effect, photodynamic therapy usually shows lower efficiency in this phase. When gold nanoring is adsorbed on the cell membrane, photothermal and photodynamic combined therapy slower the transition time [125]. Nanocarriers modified by heterogeneous ligands can control the drug release by using a pH-triggered switch. The pH-triggered switch can effectively avoid the normal tissue by realizing the H_2_O_2_ in the tumor tissue; thus, the characteristics of heterogeneous ligand-modified pH-triggered nanocarriers can selectively kill the cancer cells [126]. Singlet oxygen is recognized as cytotoxic to cells. Production of singlet oxygen in the living cell can be a trigger to cancer cell death. F. Anquez and co-workers introduced a 1270 nm laser for the production of singlet oxygen in the living tumor cells and, therefore, cell death [127]. Photothermal and photodynamic therapy have both healing effects on the tumor cells and enhance the relative attenuation of the inflammatory cells with apoptotic and necrosis tumor cells [128].

**Figure 8:**
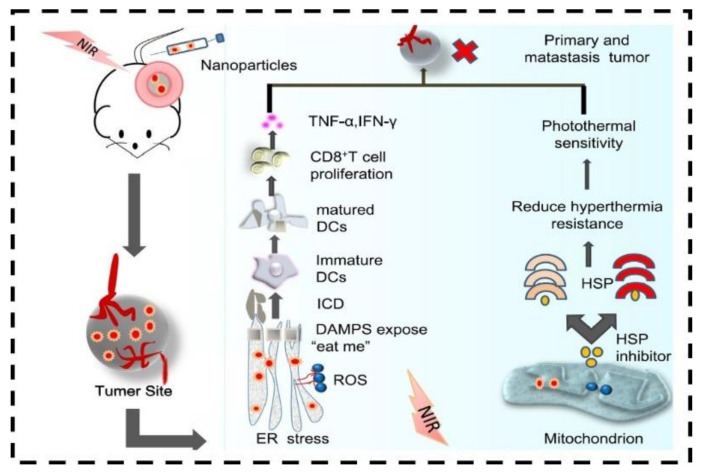
Schematic diagram of anticancer effect of the nanomaterials selective to ER and mitochondria under the NIR laser irradiation for photothermal and photodynamic therapy. Adapted with permission from Ref. [131]. 2020, Dovepress.

It is reported that the reactive oxygen species produced by endoplasmic reticulum (ER) stress in photodynamic therapy promote apoptotic cell death [129]. The main reason for immunosuppression inside the tumor is the aspect of indoleamine 2-dioxygenase generated by IFN-γ. Excess indoleamine 2-dioxygenase consumes more L-tryptophan and accumulates canine at γ; thus, it causes the inhibition of the mTOR pathway from the interference with P-S6K phosphorylation. Therefore, persuade the regulator T cell to inhibit CD8^+^ T cell activation [130]. It has been reported that CD47-targeted Ab-PEG-Bi_2_Se_3_ selectively blocks the contact between CD47 and SIRPα, hence improving the phagocytosis of macrophages resulting in enhanced photothermal therapy (Figure 8) [124,131].

HSPs are all-pervasive molecules, expressive of correct protein folding at high temperatures [132]. Some HSPs, specifically HSP60 have an anti-apoptotic role by blocking the stress kinase pathway [133]. The design for reducing the anti-apoptotic protein complex formation is to inhibit the HSP. A late study reported that cantharidin-tellurium nanoparticles (m-CTD@Te) can inhibit the anti-apoptotic proteins, thus lowering the anti-apoptotic signal to effective photothermal therapy [134].

HSP72 is also a member of the HSP family. Wang et al. also introduced an HSP72 inhibitory material indocyanine green-loaded vanadium (VO_2_-ICG) by using the layer-by-layer method, which has a similar mechanism as HSP70 [135].

## 5. Conclusions and Perspectives

Various NPs have been documented as effective light-responsive materials for selective cell behavioral control. The photothermal application of NPs can be improved by changing and tuning the dimensions of NPs, but the potential threat of these treatments is the normal cell in the body. Some biological research has shown welcome results for the NPs to control cell behavior, although it is in the early stage. On the other hand, with the promising NPs to control cell behavior under suitable laser irradiation, we may be capable of synthesizing compounds which are more effective under laser irradiation with minimal cytotoxicity. Some biocompatible coating mechanisms have been introduced to produce safe materials inside the body such as silica-coating and PEG-coating, which has been proven as a biocompatible materials coating technology for photothermal applications in vivo. However, tracking the NPs after administration is difficult. So, more technologies and tactics for monitoring and tracking the NPs after the treatment in vivo are needed to study.

In conclusion, light-responsive NPs for extensive use still needs to be continued to reduce the cytotoxic effect and the development of advanced tracking technologies. In the future, light-responsive NPs would be effective therapies to control cell behavior and anti-tumor treatment. Furthermore, a safe NIR source with safe laser power levels has been described according to the American National Standard in this article. However, the focus should be on the light-responsive NPs integration into synergic therapies. Light-responsive NPs need to develop as multifunctional NPs, such as drug carriers, contrast agents, and controlled drug release under laser irradiation. Considering the discussions in the previous section, it could implement light-responsive biocompatible NPs for cell behavior control such as abnormal cell ablation or anti-inflammation agents in a selective manner with minimal cytotoxicity.

## Figures and Tables

**Table 1:** Summary of NIR-light-responsive materials for photothermal cell treatments.

Materials	In Vivo/In Vitro	Dose/Concentration	Laser Power and Wavelength	Cell(s)	Total Treatment Time/Laser Irradiation Time	Activity	Ref.
Si-AuNRs	In vitro	83 µg/mL	NIR Laser 160 mW, 671 nm Wavelength	MDA-MB-231	48 h	Enhance the activities of HSPs. Folding the proteins in cell growth and survival	[29]
Gold nanorods–magnetic NPs	In vitro	5 mg·mL^−1^–35 mg mL^−1^	671 nm DPSS Laser, 130 mW	*E. coli*	12 min (laser irradiation time)	Bactericidal, bacteriostatic	[31]
Chit-AgNTs	In vitro	0.17 µgmL^−1^–1.71 µgmL^−1^	720–930 nm, CW laser	NCI-H460 cancer cells	24 h	Cell-membrane destruction by photothermal effect	[33]
Fe_3_O_4_-ICG@IRM	In vivo	20 mg/kg in mice	0.5–2.0 W/cm^2^	ID8 tumor in C57BL/6 mice	18 days	Vacuolar necrotic cells, apoptotic tumor cells	[34]
IONF@CuS NPs	In vitro	10 µL	0.3 W/cm^2^	hMSCs	21 days	Potential photothermal properties with no adverse biological response	[35]
Fe_3_O_4_@Dex-PGEA	In vivo	100 µL	1 W/cm^2^	Breast cancer cells	10 days	Growth reduction in the solid tumor tissue	[36]
PEG-SWCNTs	In vivo	~120 mg/mL, 100 µL	808 nm Wavelength, 76 W/cm^3^	KB tumor cells	60 days	Destruction of the solid tumor	[37]
Ppy NPs	In vivo	0.072–2.3 mg/mL	1 W/cm^2^	U87 tumor cells	18 days	Prominent photothermal efficiency with excellent biosafety	[38]
Graphene nanocomposite	In vitro	0–20%	808 nm, 800 mW	Neural stem cells	7 days	Potential photothermal properties and enhanced cell proliferation	[39]

**Table 2:** Summary of nanomaterials size effects on biocompatibility.

Materials	Methods	Size	Animals	Site of Action	Route of Administration	Toxicity	Ref.
AuNPs	Percent mortality	15–100 nm	Mouse and zebrafish	Size-dependent distribution	Intravenous (mouse), embryo	No toxicity observed	[38,39]
AgNPs	Histopathology	42 nm	Mouse	Whole body distribution	Oral	Organ toxicity and inflammatory responses	[75]
TiO_2_	Morphometric	19–21 nm	Mouse	Placenta	Intratracheal	Pulmonary toxicity, pulmonary emphysema	[76]
Nano-copper	Biochemistry analysis	~23.5 nm	Mouse	Plasma	Oral	Accumulation of alkalescent substance	[77]
Silica nanoparticles	Immunohistochemistry	50–200 nm	Mouse	Tissue distribution	Intravenous	Inflammatory responses over the size ~100 nm	[78]
PLGA	Histopathology	200–350 nm	Mouse	Histopathology assay	Oral	No toxicity	[79]
